# Using Small Molecules to Reprogram RPE Cells in Regenerative Medicine for Degenerative Eye Disease

**DOI:** 10.3390/cells13231931

**Published:** 2024-11-21

**Authors:** Lyubov A. Rzhanova, Elena V. Alpeeva, Maria A. Aleksandrova

**Affiliations:** Koltzov Institute of Developmental Biology of the Russian Academy of Sciences, 26 Vavilov Street, 119334 Moscow, Russia; mariaaleks@inbox.ru

**Keywords:** retinal pigment epithelium, RPE, reprogramming, chemically directed reprogramming, small molecules, inherited retinal degeneration, eye degeneration disease, regenerative and rehabilitation medicine

## Abstract

The main purpose of regenerative medicine for degenerative eye diseases is to create cells to replace lost or damaged ones. Due to their anatomical, genetic, and epigenetic features, characteristics of origin, evolutionary inheritance, capacity for dedifferentiation, proliferation, and plasticity, mammalian and human RPE cells are of great interest as endogenous sources of new photoreceptors and other neurons for the degrading retina. Promising methods for the reprogramming of RPE cells into retinal cells include genetic methods and chemical methods under the influence of certain low-molecular-weight compounds, so-called small molecules. Depending on the goal, which can be the preservation or the replacement of lost RPE cells and cellular structures, various small molecules are used to influence certain biological processes at different levels of cellular regulation. This review discusses the potential of the chemical reprogramming of RPE cells in comparison with other somatic cells and induced pluripotent stem cells (iPSCs) into neural cells of the brain and retina. Possible mechanisms of the chemically induced reprogramming of somatic cells under the influence of small molecules are explored and compared. This review also considers other possibilities in using them in the treatment of retinal degenerative diseases based on the protection, preservation, and support of survived RPE and retinal cells.

## 1. Introduction

Age-related macular degeneration (AMD) is the leading cause of blindness in developed countries and the third leading cause of blindness in the world [1]. Age is a significant risk factor for the development of AMD, and the prevalence of AMD is expected to increase with the increase in life expectancy. An estimated 288 million people are likely to have AMD by 2040 [2]. AMD affects the macular region of the retina and in advanced stages, it leads to the loss of central vision. One can distinguish dry (atrophic) and wet (exudative) forms of AMD. In the early stages of AMD, it is characterized by loss of the retinal pigment epithelium (RPE) and accumulation of drusen between Bruch’s membrane and the RPE layer [2]. The late stage is characterized by geographic atrophy with degeneration of the RPE and loss of photoreceptors, which depend on the RPE for trophic support. Wet AMD manifests as choroidal neovascularization, in which new blood vessels grow into the retina, leading to destruction of the RPE and photoreceptors [1]. Although vascular endothelial growth factor (VEGF) inhibition has shown promising results in the treatment of wet AMD, currently, there is no treatment for atrophic AMD [2].

While AMD primarily affects elderly people, retinitis pigmentosa often appears in the first years of life. Retinitis pigmentosa is a heterogeneous group of genetic diseases affecting 1 in 3000–7000 people. Patients with retinitis pigmentosa experience severe visual impairment at the age of 40–50 [3]. Degeneration of rods with subsequent loss of cones in the retina is the hallmark of retinitis pigmentosa. Typical symptoms of retinitis pigmentosa are night blindness and tunnel vision, which eventually lead to complete blindness. These disorders and other degenerative retinal diseases are virtually impossible to repair with existing treatment methods. In this regard, there is an active search for new ways to regenerate visual structures, among which the cell replacement strategy has high potential.

Currently, two strategies can be conveniently distinguished: regenerative medicine and molecular medicine. Regenerative medicine aims to restore normal function by replacing or regenerating human cells, while the focus of traditional molecular medicine has long been on using small molecule medicines (small molecules). However, in recent years, many attempts have been made to search for small molecules for particular use in regenerative medicine, taking into consideration the possibility of their pharmacological control and usability [4].

Small molecules are chemical compounds with a molecular weight between 500 and 900 Daltons. They have biological activity and can regulate or influence certain biological processes. At the cellular level, small molecules can change genetic, epigenetic, and protein levels by influencing signaling pathways and regulating cell activity, function, and identity. Depending on the goal, to protect, preserve, and maintain remaining cells or to replace lost ones during the progression of degenerative eye disease RPE and retinal cells, various small molecules are used, acting at different levels of cellular regulation. This review examines the potential of small molecules to reprogram mammalian and human RPE into neuronal cells of the retina and brain, comparing this with the neuronal reprogramming of other somatic cell types and induced pluripotent stem cells (iPSCs). This possibility of chemical reprogramming under the influence of small molecules is especially interesting from the point of view of endogenous regeneration of damaged RPE and photoreceptors using the eye’s own RPE cells, which avoids many problems associated with transplantation. RPE cells of the vertebrate and human eye have a number of features that allow them to be considered valuable endogenous sources of cells for replacement therapy [5,6].

RPE cells have limited plasticity, which can be affected by external stimuli [6,7,8]. This limitation is mainly due to epigenetic factors, which do not allow full reprogramming of RPE into retinal cells, even with the help of the ectopic expression of genes encoding neuron-specific transcription factors (TF) [8,9,10,11]. At the same time, chemical reprogramming of RPE demonstrates great success in obtaining retinal neurons. The use of small molecules affecting the epigenome, TGFβ-, GSK3β-, WNT-, and BMP-signaling pathways was shown to reprogram RPE cells into rod photoreceptors [12]. To understand the mechanisms of the chemical reprogramming of mammalian and human RPE cells into retinal neurons, they are compared with the mechanisms of chemical reprogramming of other somatic cells in this review. Despite the fact that the chemically induced reprogramming of RPE cells is a new direction and, thus, poorly studied, it demonstrates certain results that give great hope for success and the possibility of treating degenerative eye diseases with easily accessible pharmacological agents.

## 2. Regeneration of RPE and Retinal Cells via RPE Reprogramming with Small Molecules

Progress in the application of cell replacement technologies for vision restoration in degenerative eye diseases is reviewed in detail in [13]. The authors discuss clinical trials of RPE transplantation, advanced preclinical research on the transplantation of other cell types, prosthesis, opsin-based gene therapy (“optogenetics”), and small molecule photoswitches. They also discuss progress in the in situ restoration of deteriorating retina using endogenous progenitor cells, Müller glia cells [13], but in our work, we would like to draw attention to another source of endogenous RPE cells.

### 2.1. The RPE as an Important Endogenous Cell Source in Regenerative Medicine

The RPE represents a monolayer of hexagonal, pigmented, polarized cells that are located between the neural retina and the choroid. RPE cells perform a number of important functions necessary for maintaining the structural integrity of the choriocapillaris endothelium and photoreceptors. These functions include restoration of the visual chromophore 11-cis-retinal, phagocytosis of shed photoreceptor outer segments, the formation of the blood–retinal barrier, transport of nutrients to the photoreceptors, regulation of ion, pH, and fluid homeostasis in the subretinal space, absorption of light energy, free radicals and reactive oxygen species scavenging, and secretion of various growth factors [14]. The neural part of the retina and the RPE develop from the same neuroepithelial progenitor cells under the influence of different factors [15].

Mammalian and human RPE cells are of great interest as sources of new photoreceptors and other neurons in the degenerating retina, taking into account their anatomical, genetic, and epigenetic characteristics, origin, evolutionary heritage, and ability to dedifferentiate, proliferate, and be flexible [16].

**Figure 1:**
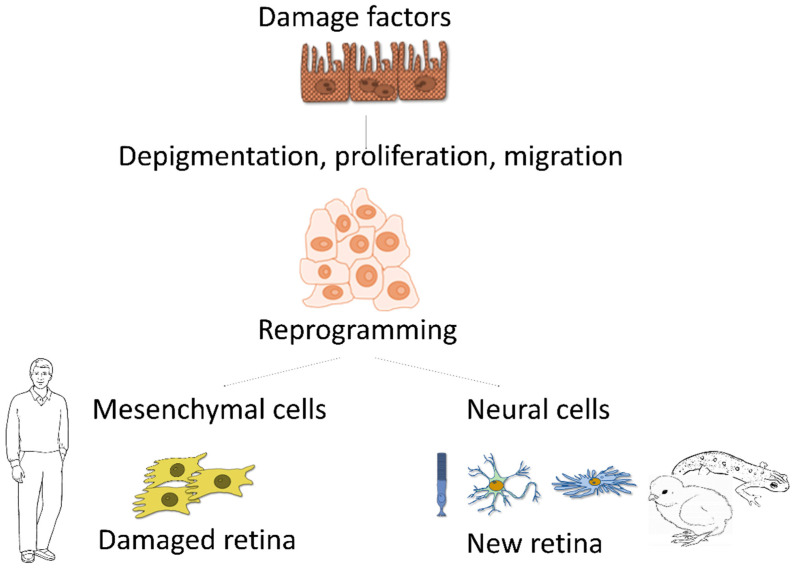
Eye injury induces different RPE responses in lower vertebrates and mammals, including humans. The initial stages of cellular reprogramming are the same in all studied organisms: cells enter the cell cycle, begin to proliferate, lose pigment, and dedifferentiate. However, subsequently, the cells develop completely differently: in some species, RPE cells are transformed into neuronal retinal cells, thereby restoring the retina, while in humans, dedifferentiated RPE cells differentiate into myofibroblasts, which leads to serious pathologies.

RPE cells exhibit a wide range of plasticity in different contexts. For example, in lower vertebrates, they can generate retinal neurons. When damaged or under retinectomy, RPE cells activate dedifferentiation processes, which are based on the disruption of intercellular contacts, loss of pigmentation, cell cycle resumption, proliferation, cell migration, and expression of proteins that are not characteristic for the RPE, which leads to a change in the cell phenotype [17,18]. However, unlike lower vertebrate RPE cells, which undergo retinal repair through transdifferentiation, human RPE cells undergo epithelial-mesenchymal transition (EMT) and transform into spindle-shaped cells resembling fibroblasts and macrophages (Figure 1). These cells migrate into the subretinal space and toward the vitreous body, where they participate in the formation of neovascular epiretinal membranes, thereby damaging the retina and other structures of the eye, and this causes visual impairment and blindness [17,19,20,21]. However, a number of researchers consider this behavior of human and mammalian RPE cells to be a residual ability of RPE cells to transdifferentiate into retinal cells, which was limited by changes in the regulation of the transdifferentiation process and the patterns of rapid scar regeneration in the course of evolution [22]. The modification of the molecular mechanism for successful neural regeneration observed in Urodela was proposed to underlie RPE-dependent retinal pathologies in mammals [23].

An analysis of the events and processes occurring during reprogramming of RPE cells in different species, amphibians, birds, and mammals, including humans, has shown a conserved pathway for the induction and control of cellular transformation [22]. Thus, the mammalian RPE was demonstrated to possess the properties necessary for induction and transformation into the retina: proliferative activity and loss of pigmentation. During proliferation, mammalian RPE cells use the same mechanisms of entry into the S-phase in vitro as RPE cells of amphibians and birds in vivo, in which FGF2 and MAPK and ERK kinases play a key role. The phosphorylation of MAPK/ERK in turn increases the expression of the “developmental” genes *c-Myc*, *Pax6*, *Klf4*, *Mitf*, etc., indicating a decrease in the level of differentiation of RPE cells [24,25]. These intracellular events are initiated immediately after RPE cells leave the epithelial layer in amphibians and birds in vivo. Next, the dedifferentiated cells implement the already established choice of development towards neurons or mesenchyme. While RPE cells of amphibians and birds begin to differentiate along the neural pathway to restore the damaged retina, in mammals and in humans, RPE cells enter the EMT and transform into myofibroblasts. Initially almost identical in their potential RPE cells, in mammals and amphibians end up in different microenvironments, which regulate the divergence [22]. Understanding regulation by the cellular microenvironment is a key to targeting RPE cell behavior to restore the retina and diminish pathological conditions.

The microenvironmental factors involved in the transdifferentiation of RPE cells into retinal cells are largely similar to developmental factors in their range and nature [22]. Among the signaling cascades involved in the control of neural retina regeneration in amphibians, FGF, BMP, WNT, SHH, and Notch signaling are the most important [26]. In mice, increased expression of multiple growth factors such as Vegf, Tgf, Pdgf, Egf, and Ngf and their receptors, Vegfr, Egfr, Pdgfr, and Tlk4, as well as two stem-cell-associated signaling ligands, Kitl and Lif, was demonstrated. Their role in inducing RPE cell rejuvenation was discussed [27]. The authors demonstrated the crucial role of Wnt and Hippo signaling in the reprogramming of mouse RPE cells [27]. The role of fibroblast growth factor (FGF2), which plays a key role in successful retinal regeneration, has been most thoroughly studied in tailed amphibians and chicken embryos [28,29,30,31]. One of the approaches to enhancing the regenerative capacity of the mammalian and human RPE may involve modulating proregenerative signaling pathways that are either inactive in injured mammalian RPE or only moderately activated by injury [32]. These changes in the signaling pathways stimulate proliferative activity, restore the expression pattern of developmental genes and multipotency genes (i.e., the conditions of “rejuvenation”), and provide control over further development.

The dedifferentiation mechanism is triggered in RPE cells under certain conditions, such as with the loss of cellular contacts. This process is accompanied by depigmentation, entry into the cell cycle, proliferation, and the expression of multipotency and neural stem cell genes [7]. Whereas the introduction of Yamanaka factors or stimulation with small molecules is required to obtain iPSCs from fibroblasts [33], the expression of these TFs is a natural property of RPE cells themselves. During dedifferentiation, RPE cells express OCT4 (POU5F1), KLF4, SOX2, C-MYC, and NANOG [7], which are the most important regulators of pluripotency and allow for the induction of the reprogramming of somatic cells into iPSCs. This means that RPE cells themselves can be reprogrammed into the NSC state (i.e., dedifferentiated), especially in vitro. However, it is necessary to control the expression of OTX2, which is an antagonist of the dedifferentiation process in mammalian and human RPE cells themselves, as its increased expression returns dedifferentiated RPE cells to a differentiated state [5]. From this point of view, when reprogramming RPE cells into retinal cells, as well as for iPSCs and ESCs, under experimental conditions, using the VCR cocktail as an epigenetic modulator is not necessary. It is most likely that RPE cells can be directly reprogrammed into retinal cells, such as photoreceptors, in the same way as astrocytes into CiNs, using chemical cocktails containing CHIR99021 and RepSox [34].

Therefore, by understanding the role of the transcriptional regulators and signaling pathways involved in these processes and the principles of cellular reprogramming, it is possible to obtain the desired reprogramming of RPE cells into retinal neurons.

### 2.2. Chemical Reprogramming of Cells Under the Influence of Small Molecules

Cell fate decisions are typically controlled by TFs that regulate gene expression in association with epigenetic modifications. Altering information through the regulation of gene expression levels and/or epigenetic modification results in the reprogramming of one cell into another [35]. However, a comprehensive study of the world literature revealed that it is impossible to obtain full-fledged retinal cells from the RPE of an adult human using direct genetically mediated reprogramming, which is aimed at the ectopic expression of specific TFs. Many researchers have suggested that the RPE of mammals and humans has a highly developed epigenetic system for controlling cell identity [8,9]. An alternative method for genetic reprogramming of cells is chemical reprogramming with small molecules.

#### 2.2.1. Small Molecules Used for Chemically Induced Reprogramming of Different Cell Types into Neural Cells

Small molecules are chemical compounds with a molecular weight between 500 and 900 Daltons. They have biological activity and can regulate or influence certain biological processes. The strategy of chemical reprogramming is based on the influence of small molecules on the epigenome, signaling or metabolic pathways, which ultimately affect the activity of TFs that determine cell fate (Table 1).

Today, a number of cells, including pluripotent stem cells, neural progenitors, neurons, and cardiomyocytes, can be obtained from terminally differentiated cells under the influence of small molecule cocktails in vivo and in vitro [33,55,56]. The principle of chemical or low-molecular reprogramming is the same for any cell. Most small molecules can be divided into several groups: epigenetic modulators, molecules acting on different cell signaling pathways, among which are modulators of epithelial–mesenchymal and mesenchymal–epithelial transitions, metabolic regulators, regulators that promote the self-renewal of ESCs, etc. (Table 1).

#### 2.2.2. Epigenetic Modulators

Epigenetic marks such as the acetylation of histones and the methylation of histones or DNA serve to induce or inhibit gene expression in a heritable manner. Global changes in epigenetic marks are critical for reprogramming [35]. Histone deacetylase (HDAC) inhibitors, such as VPA, NaB, and TSA (trichostatin A), are used as the epigenetic factors stimulating gene transcription due to hyperacetylation, which leads to an increase in the gap between the nucleosome and the DNA wound around it. Small molecules like Bix01294 (histone methyltransferase inhibitors) target the enzymes that methylate and demethylate histones and raise the expression levels of KLF4 and Oct4 [33]. In combination with a glycogen synthase kinase 3 (GSK3β) inhibitor CHIR99021, a lysine-specific demethylase inhibitor (LSD1) mediates H3K4 demethylation, which allows for the induction of KLF4 and OCT4 in being reprogrammed into iPSCs human keratinocytes. Epigenetic modulators are necessary for cellular "rejuvenation" to make reprogrammed cells enter the cell cycle and begin proliferation [33]. At this stage, a transient population of cells with plasticity is formed.

Cellular rejuvenation through partial reprogramming has been shown to be a promising way to achieve the goals of regenerative medicine, as it targets the loss of epigenetic information during aging and injury [57,58]. The loss of epigenetic rather than genetic information has been implicated as a potential cause of aging. It has been shown that DNA age and gene expression patterns in aged and damaged neurons can be safely reversed by ectopic expression of the DNA demethylases TET1 and TET2 [59]. These results demonstrated that cells have a “backup” copy of young epigenetic information that can restore cell identity [58]. To achieve age reduction without altering cell identity, researchers analyzed the action of 80 small molecule chemical cocktails. Among those tested in the nucleocytoplasmic compartmentalization (NCC) assay, the basal VC6TF cocktail was the most effective in restoring integrity, a key feature of aging reversal. This cocktail consisted of VAP, CHIR-99021, E-616452 (RepSox), tranylcypromine, and forskolin [56]. Forskolin is an adenylate cyclase activator that has been shown to stimulate reprogramming and transdifferentiation, depending on the combination of other compounds present. Although the mechanism of action of forskolin is controversial, its action may increase cellular cAMP levels and initiate signaling cascades that are critical for the adoption of cellular identity [56].

When a somatic cell is reprogrammed into CiPSC, it has to acquire the characteristics of ESCs, the ability to self-renew. It is necessary to “rejuvenate” the somatic cell to stimulate proliferation, which is usually limited in somatic cells [33]. The researchers note that compared to ESCs, somatic cells have a much more compact epigenome, which results in the epigenetically suppressed expression of ESC-specific genes. Therefore, derepression of the pluripotency circuit is required to generate iPSCs [33]. For this stage, the VCR cocktail is used, which is a powerful epigenetic regulator that relieves epigenetic “tension” in reprogrammed fibroblasts and rejuvenates cells [56]. This stage can also be observed during the reprogramming of terminally differentiated somatic cells into any other cell type when the reprogrammed cells pass through a transient population close to ESCs, which represent poorly differentiated progenitor proliferating cells [60].

Studies of developmental epigenetics have shown that DNA demethylation plays an important role during neurogenesis. During development, the methylation of the genes associated with photoreceptors and phototransduction was observed in the RPE, which disappears in the differentiating photoreceptor progenitor [8]. The authors who made this observation suggest that methylome dynamics and TET demethylase activity are crucial for neurogenesis and retinal development. It should be noted that the expression of Tet1-3 (ten-eleven translocation (TET) family enzymes) is very low in the RPE [8,60]. Increased expression of all the genes in the TET and DNMT3 families was shown, revealing their critical role in altering the DNA methylation landscape of the human CiPCs derived from the fetal RPE [12]. Therefore, the forced activation of TET genes or the use of RG108, a DNA methyltransferase inhibitor, in the adult RPE may stimulate photoreceptor phenotypes in these cells [8]. To achieve this, hypoxic conditions can be created, which causes the hyperexpression of TET 1 and 2 in RPE cells [61]. Vitamin C has been shown to help remove repressive epigenetic marks from gene promoters through the regulation of histone demethylases and TET proteins, thereby reducing gene expression. This facilitates a faster reprogramming process that produces iPSCs that are uniform and of a higher quality [33]. Shen et al. successfully dedifferentiated RPE cells into stem cell status using VPA and vitamin C [46]. There is evidence that HDAC is not involved in the aging process of RPE cells [62], whereas other enzymes, such as TET demethylases, are important for it.

#### 2.2.3. Molecules Acting on Signaling Pathways

**Figure 2:**
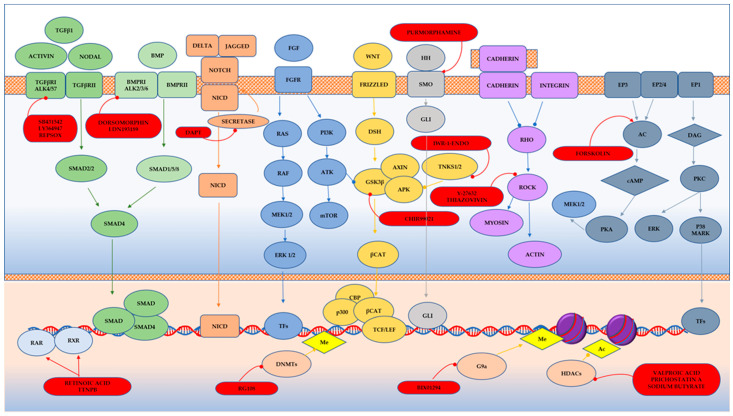
Some small molecules and signaling pathways promoting the development of the nervous system (based on the poster https://www.stemcell.com/media/files/wallchart/WA10014-Small_Molecules_Big_Impact.pdf (accessed on 16 November 2024)). Notes: The transforming growth factor β (TGFβ) is involved in a whole range of biological functions, from cell growth to cell differentiation and apoptosis. SMAD1, 2, 3, 5, and 8 are receptor-regulated SMADs. They bind to membrane-bound serine/threonine receptors and are activated by the kinase activity of the receptors. SMAD4 acts as a cofactor that binds to activated R-SMADS (SMADs) forming a complex that translocates into the nucleus [63]. Pathway inhibitors: SB431542, LY364947, RepSox, Dorsomorphin, LDN193189. The Notch signaling pathway regulates cell proliferation, cell fate, differentiation, and cell death in all metazoans. The Notch pathway is activated when Delta or Jagged ligands on neighboring cells activate cleavage of the receptor releasing the Notch intracellular domain (NICD). The Notch pathway plays a role in specifying neural subtypes [64]. Pathway inhibitors: DAPT, LY411575. Fibroblast growth factor (FGF) signaling regulates several developmental processes, including cellular proliferation, differentiation, migration, morphogenesis, and patterning. FGF signaling via MEK/ERK is critical for self-renewal and proliferation of human PSCs [65]. The WNT signaling pathway is an ancient and evolutionarily conserved pathway that regulates crucial aspects of cell fate determination, cell migration, cell polarity, neural patterning, and organogenesis during embryonic development [66]. Pathway activators: CHIR99021, SB216763; pathway inhibitors: IWR-1-endo. The Hedgehog (Shh) pathway is important in post-embryonic tissue regeneration and repair processes. Specifically, Shh signaling is implicated in the induction of multifarious neuronal populations in central nervous system [67]. Pathway activators: Purmorphamine, SAg. The RHO/ROCK pathway regulates cytoskeletal dynamics and plays an important role in cell adhesion, proliferation, motility, contraction, and apoptosis. Loss of cadherin or integrin binding activates the Rho pathway in human PSCs, leading to anoikis [68]. Pathway inhibitors: Y-27632, thiazovivin. The 3′,5′-cyclic adenosine monophosphate (cAMP) is a second messenger important in reprogramming and differentiation for many cell subtypes [69]. Pathway activator: forskolin. The protein kinase C (PKC) family of kinases is commonly activated by diacylglycerol (DAG) and calcium and is involved in several signaling pathways that can regulate differentiation [70]. Pathway activators: prostaglandin E2, (−)-Indolactam V; pathway inhibitors: HA-100, GO6983. Retinoic acid (RA) is a potent morphogen required for embryonic development. RA acts in a paracrine fashion to shape the developing eye and is essential for normal optic vesicle and anterior segment formation [71]. Activators: 9-cis retinoic acid, all-trans retinoic acid, CD437, TTNPB. RAR, RXR -RA receptors. Epigenetic marks such as acetylation (Ac) of histones and methylation (Me) of histones or DNA serve to induce or inhibit gene expression in a heritable manner. Global changes in epigenetic marks are critical for reprogramming [35]. DNA Methyltransferase inhibitors: RG108; histone methyltransferase inhibitors: BIX01294; histone demethylase inhibitors: tranylcypromine; histone acetyltransferase inhibitors: garcinol; histone deacetylase inhibitors: sodium butyrate, trichostatin A, valproic acid.

Small molecules can suppress the initial differentiation of a cell and activate another direction of the differentiation by influencing signaling pathways (Figure 2).

**Molecules that suppress the original characteristics of the cells.** For example, if the original cell has a mesenchymal phenotype, it is necessary to induce MET in it. For this purpose, an inhibitor of Wnt and TGF signaling pathways, an inhibitor of GSK3β CHIR99021 is usually used. It has been shown that inhibition of four signaling pathways by small molecules Notch, TGF-β, BMP, and GSK-3β is sufficient for efficient reprogramming of human fetal glial cells into neurons in vitro and for activation of neurogenesis in the mouse hippocampus in vivo [50]. Zhang et al. used LDN193189, SB431542, and TTNPB to inhibit glial signaling pathways and activate neuronal signaling pathways and thiazovivin (Tzv) to improve reprogramming efficiency and cell survival [38,72]. When differentiating ESCs and iPSCs into RPE cells, most protocols initially involve inhibition of TGF-β and Wnt signaling pathways by small molecules, followed by their activation [73].

**Compounds that induce development of the characteristics of the resulting cells**. Small molecules used to reprogram cells into CiPSCs, CiNSCs, and CiNs activate the Wnt signaling pathway and inhibit TGF-β and BMP signals. The Wnt signal is known to stimulate neurogenesis, and inhibition of TGF-β and BMP signals induces differentiation of pluripotent cells [72]. For the induction of neurons, compounds are required that will promote the specialization and maturation of neurons, such as ISX9 and Dorsomorphin [12,37,43]. For reprogramming astrocytes into neurons, CHIR99021 and DAPT were used to induce CiNs, followed by treating the cells with smoothened agonist and purmorphamine to activate the Sonic Hedgehog signaling pathway, which is the main factor in neural patterning [38]. The presence of forskolin, noggin, retinoic acid, and a number of growth factors important for neurogenesis (bFGF and IGF) is required for successful and effective reprogramming of RPE cells into neural retinal cells.

#### 2.2.4. Mesenchymal–Epithelial Transition

Fibroblasts and ESCs have different cellular morphologies: fibroblasts have a mesenchymal morphology, and ESCs have an epithelial morphology [33]. Thus, when reprogramming fibroblasts into iPSCs, it is necessary to initiate MET [33]. For its induction, the inhibitors of Wnt and TGF signaling pathways and the inhibitor of GSK3 CHIR99021 are used. For RPE reprogramming, MET induction is not required, as RPE cells are epithelial cells. However, it is necessary to initiate controlled EMT so that RPE cells can leave the monolayer. RPE cells must be restrained from further EMT, which mammalian and human RPE cells usually strive for, to follow the differentiation direction required by the researcher. It has been shown that nicotinamide (NAM) enhances the RPE phenotype and prevents EMT in several model systems of RPE cells [74,75,76]. Thus, in adult human stem cell-derived RPE cell cultures, RPESC-RPE NAM prevents and reverses EMT induced by TGF-β and TNF-α exposure [77]. The possible mechanisms by which NAM promotes RPE cell survival and differentiation include the inhibition of Rho-associated protein kinase (ROCK) and casein kinase 1 (CK1) [75]. Thus, it was shown that the use of AKT inhibitors, AKT inhibitor IV and ERK inhibitor U0126, leads to a decrease in the expression of Snail and N-cadherin, as well as CTGF and fibronectin, and therefore to a decrease in EMT in RPE cells [78]. In the chemical reprogramming of the RPE, Wnt (DKK1 and XAV939) and BMP (Noggin and dorsomorphin) inhibitors are used [12,46].

#### 2.2.5. Metabolic Shift from Mitochondrial Energy to Glycolysis

Even under normal oxygen conditions, ESCs rely primarily on enhanced glycolysis, whereas somatic cells use mitochondrial oxidative phosphorylation (OXPHOS) to produce ATP. Neuronal progenitor cells in the embryonic brain also use glycolysis to produce ATP, whereas differentiated neurons use OXPHOS [79]. Glycolytic metabolism is known to play an additional role in supporting mitotic capacity and RPC survival that is unrelated to energy production. Glycolytic metabolism is directly regulated by mTORC1 signaling, and this is also the case in the retina. A metabolic shift from mitochondrial energy to glycolysis is required to facilitate reprogramming and contribute to the global epigenetic landscape [33]. Programmed mitophagy, or mitochondria-directed autophagy, occurs in several developing tissues, causing metabolic reprogramming from OXPHOS to glycolysis [80]. Sodium butyrate, a histone deacetylase inhibitor, and other small molecules (PD0325901 and A-83-01) were sequentially used in combination with PS48, an activator of 3′-phosphoinositide-dependent protein kinase 1, to reprogram amniotic fluid-derived cells, adult keratinocytes, and umbilical vein endothelial cells. PS48 enhances gene expression, promotes glycolytic metabolism and mitochondrial oxidative phosphorylation, and activates the phosphoinositide 3-kinase/Akt pathway [33,81].

#### 2.2.6. Factors That Promote the Survival and Functioning of Reprogrammed Cells

Factors that promote the survival and functioning of reprogrammed cells in vitro, as well as facilitating reprogramming, such as Y-27632 [37,50], enhance survival and facilitate the reprogramming of mouse fibroblasts into neurons at an early stage of induction. One can also use the ROCK inhibitors, Y27632 and Fasudil, or the P38 and MAPK inhibitors, SB203580 and BIRB796 [43]. Growth factors, such as bFGF, IGF1, BDNF, and GDNF, are implemented for the maturation of induced neural cells in addition to small molecules [38,43].

Compared with other methods, reprogamming with small molecules have a number of unique advantages, such as versatility of chemical structure and ease of manipulation, depending on time and concentration. Small molecules are non-immunogenic, cell-permeable, cost-effective, and easy to synthesize, store, and standardize. In addition, the effect of using small molecules is reversible. Cells modified with small molecules are more technologically advanced than cells modified using alternative genetic approaches, and their activity can be limited in space and time, providing an additional level of safety in clinical applications [46]. This method of direct reprogramming has great promise due to a number of advantages. Today, chemically mediated cellular reprogramming is being actively studied and developed, and researchers around the world are trying to find an adequate replacement for TFs transfection with chemical molecules. Reprogramming can be even more effective through the action of small signal molecules in combination with the introduction of specific factors [42]. Recently, increasing attention has been paid to microRNAs (miR-9 and miR-124) and their regulatory networks as potential drivers of neural transdifferentiation [82,83].

### 2.3. Chemically Induced Cell Reprogramming into CiNSCs and CiNs

Small molecule experiments began by combining the exogenous expression of TFs with small molecules to increase the efficiency of TF-based direct reprogramming. Over the past few years, significant advances have been made in small molecule approaches to inducing pluripotent or functionally differentiated cells from somatic cells [42,55]. Thus, a combination of chemical compounds and one transcription factor was shown to be sufficient to reprogram a somatic cell into iPSCs in 2010 [81]. Subsequently, it was discovered that a combination of just seven small molecules was sufficient to chemically reprogram somatic cells into iPSCs [84]. These “chemical” iPSCs were similar to ESCs in terms of gene expression profile and epigenetic state and did not require the exogenous expression of pluripotency genes [84].

There is a universal algorithm for the chemical reprogramming of any cell: first, one acts on the epigenome, thereby “rejuvenating” the cells or bringing them to the state of iPSCs, then influences the signaling pathways that regulate differentiation in one direction or another. That is why a typical small molecule cocktail used to reprogram any cell includes the following components: epigenetic modulators, molecules that suppress the original characteristics of the cells, compounds that induce the characteristics of the resulting cells, and factors that promote the survival and functioning of the reprogrammed cells in vitro [42] (Table 2).

#### 2.3.1. Reprogramming of Fibroblasts into CiNSCs and CiNs

**Figure 3:**
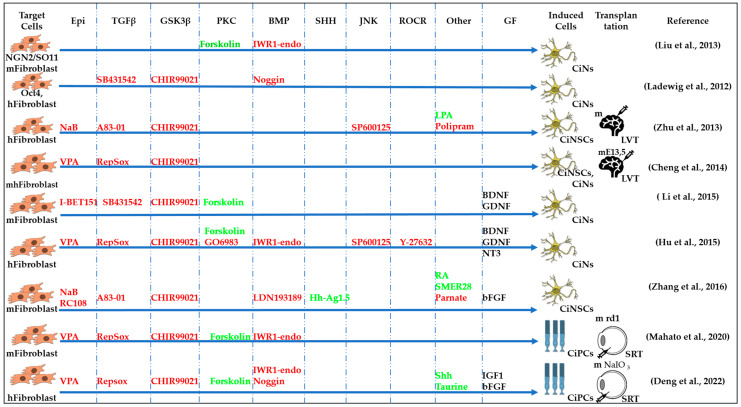
Schematic representation of chemically induced reprogramming of fibroblasts into neural stem cells and into neurons in the brain and retina [12,36,37,39,41,42,43,85,86]. This approach utilized small molecules that acted on the cellular epigenome (Epi) and on various signaling pathways that control cellular identity (TGFβ, GSK3β, PKC, BMP, SHH, JNR, ROCK, and others). FG—growth factor; m—mouse; h—human; CiNs—chemically induced neurons; CiNSCs—chemically induced neural stem cells; CiPCs—chemically induced photoreceptor-like cells; m rd1—mouse model of retinal degeneration; m NaIO_3_—mouse model of sodium iodate (NaIO_3_)-induced retinal degeneration; SRT—subretinal transplantation, LVT—lateral ventricle transplantation. Pathway activators: green color; pathway inhibitors: red color.

Induced neural stem cells (iNSCs) were generated using neuronal-lineage-specific TFs, such as SOX2. The resulting cells were multipotent and could differentiate into functional neurons, astrocytes, and oligodendrocytes, both in vitro and in vivo [94,95]. Soon, both mouse and human iNSCs were generated using the CASD strategy [39]. The CASD strategy utilizes transient exposure of somatic cells to pluripotency factors (Oct4, Sox2, Klf4, and c-Myc) (cell activation, CA) in combination with soluble-tissue-specific signals targeting cell signaling (signaling-directed, SD) to reprogram cells into other cell types, such as iNSCs. The authors created a chemical cocktail containing A83-01, CHIR99021, NaB, LPA, Rolipram, and SP600125 that, when combined with ectopic expression of OCT4, could transform adult human fibroblasts into chemically induced neural stem cell (CiNSCs) colonies that uniformly expressed PAX6 [39]. Other researchers have achieved more efficient reprogramming of mouse fibroblasts into CiNSCs using a cocktail of nine components: CHIR99021, LDN193189, A83-01, retinoic acid, Hh-Ag1.5, RG108, Parnate, SMER28, and bFGF (Figure 3) [41]. They hypothesized that treating cells with a combination of small molecules that target epigenetic modifications and modulate signals associated with neural development could induce a neural transcriptional program in fibroblasts. To begin a combinatorial chemical screen, LDN193189 and A83-01, which inhibit mesoderm and endoderm specification, and CHIR99021 and basic fibroblast growth factor (bFGF), which promote neural system development, were combined as the primary neural induction conditions in a chemically defined medium. Other small molecules were added for the purpose of inducing neural reprogramming in fibroblasts on top of these factors [41].

Forskolin, an activator of adenylate cyclase, which induces cAMP increase in the PKA signaling pathway, and dorsomorphin, an inhibitor of BMP, both regulate signaling pathways involved in neurogenesis (Figure 2). In a study by Liu et al., NGN2 or SOX11, along with small molecules, forskolin and dorsomorphin, directly reprogrammed human fetal lung fibroblasts into cholinergic neurons with functional electrophysiology [85]. Inhibition of SMAD as a result of inhibition of TGFβ signaling by SB431542, BMP signaling by Noggin, and GSK3β signaling by CHIR99021 have been shown to promote neuronal reprogramming (Figure 2) [43]. The first generation of fully chemically induced neural progenitor cells (CiNPCs) derived from fibroblasts was reported in 2014 by Cheng and colleagues [36]. For this purpose, the authors used three molecules: VPA, CHIR99021, and RepSox (Table 1 and Table 2). They also showed that similar cocktails consisting of HDAC, GSK3, and TGF-β inhibitors have the same effect. Using this method, chemically induced neurons (CiNs) were obtained from mouse and human fibroblasts [37,42]. The authors demonstrated that the use of five small molecules (ISX9, SB431542, forskolin, CHIR99021, and I-BET151) was sufficient to rewrite the fibroblast-specific transcriptome, activate neuron-related genes, and efficiently convert fibroblasts into CiNs with up to 90% efficiency in 16 days. The authors hypothesized that I-BET151, an inhibitor of a BET bromodomain, suppresses the fibroblast-specific program, and ISX9 activates the expression of endogenous neurogenic transcription factors that synergistically promote neuronal conversion [42]. Subsequent work by Hu et al. extended this all-chemical approach to the neuronal reprogramming of human fibroblasts using a cocktail of seven small molecules, VPA, CHIR99021, RepSox, forskolin, Sp600125, GO6983, and Y27632, and additional neurotrophic factors (BDNF, GDNF, and NT3) [37]. This reprogramming method yielded fully mature and functional CiNs containing a mixture of GABAergic, cholinergic, and dopaminergic cells and provided an alternative approach to reprogramming neurons without the aid of transcription factors. Zhang et al. generated neural stem cells from human fibroblasts using a small molecule cocktail consisting of nine components (Figure 3, Table 2). These small molecules acted in a similar way in mouse and human fibroblasts: they inhibited glial but activated neuronal signaling pathways through epigenetic and transcriptional modulation. Remarkably, these human CiNs were functional and could survive for more than 5 months in cell culture [36,41,42]. A cocktail of five small molecule factors containing VPA, CHIR99021, RepSox, forskolin, and IWR1-endo was shown to be able to chemically induce fibroblasts into chemically induced photoreceptor-like cells (CiPCs) that functionally restored pupillary reflex and visual function after transplantation into the subretinal space of mice with retinal degeneration. However, different fibroblast cell types showed variable and low conversion efficiency (0 to 1%) [86]. The authors showed that mROS-mediated NF-κB activation directly regulates Ascl1 expression and fibroblast reprogramming into CiPCs, and that mitochondria–nucleus signaling acts as a mediator for direct chemical reprogramming [86]. Despite the low conversion efficiency, the authors anticipate that optimization of the protocol may be useful for obtaining large numbers of CiPCs. For example, the temporal modulation of IWR1 in the protocol resulted in a significant increase in human adult dermal fibroblast conversion into CiPCs. Overall, fibroblast-derived photoreceptor-like cells are promising candidates for cell replacement and may provide scalable therapies for vision restoration [86].

#### 2.3.2. Reprogramming of Astrocytes into CiNSCs and CiNs

**Figure 4:**
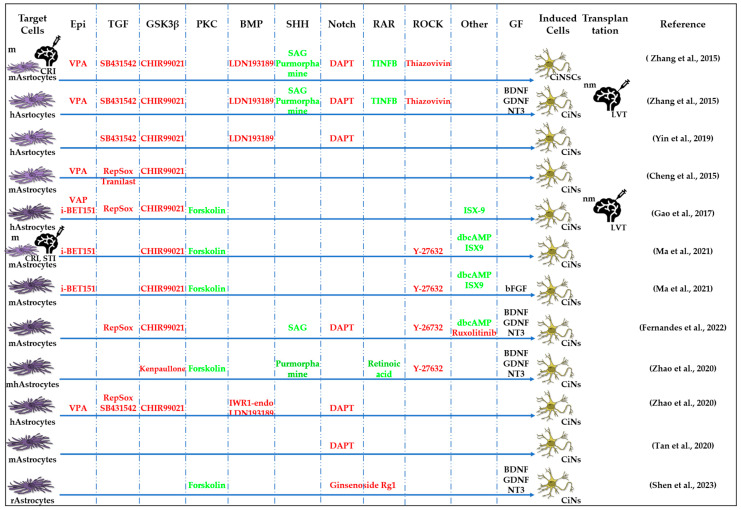
Schematic representation of chemically induced reprogramming of astrocytes into neural stem cells and into neurons in the brain and retina [34,38,50,87,88,89,90,91,92]. Chemically induced reprogramming utilized small molecules that acted on the cellular epigenome (Epi) and on various signaling pathways that control cellular identity (TGFβ, GSK3β, PKC, SHH, Notch, RAR, ROCK, and others). FG—growth factor; m—mouse; nm—neonatal mouse; h—human; r—rat; CiNs—chemically induced neurons; CiNSCs—chemically induced neural stem cells; CRI—microinjection into the cortices; STI—microinjection into the striatum; LVT—lateral ventricle transplantation. Pathway activators: green color; pathway inhibitors: red color.

Another group of researchers reprogrammed astrocytes into CiNs using a cocktail of 20 molecules that inhibit glial signaling pathways, activate neuronal signaling pathways, and are capable of epigenetic modulations at first (Figure 4). Next, they created a cocktail of nine components, LDN193189, SB431542, TTNPB, thiazovivine, CHIR99021, VPA, DAPT, as well as SAg and purmorphamine [38]. The same chemical cocktail protocol, when injected into the cerebral cortex of a neonatal mouse, reprogrammed astrocytes into CiNSCs, which produced neurospheres in culture and gave rise to neurons, astrocytes, and oligodendrocytes. After implantation into the mouse brain, the induced neurons not only survived but also were able to integrate into the neural network of the host [38]. In 2015, Cheng et al. used a mixture of small molecules, VPA, CHIR99021, and RepSox, to directly convert mouse astrocytes into functional CiNs [34]. The efficiency of transformation was improved by adding ISX-9, I-BET151, and forskolin. The transformed neurons remained viable when transplanted into mice [87]. The protocol was then optimized by adding DBcAMP and Y27632 [88]. Many researchers have noted that chemical reprogramming causes the activation of neural transcription factors, such as neurogenic differentiation 1 (NeuroD1) and neurogenin-2 (NGN2), which then continue the process of cellular transformation [41,49]. Fernandes et al. screened a six-chemical cocktail (6C) consisting of Y26732, DAPT, RepSox, CHIR99021, ruxolitinib, and SAg. In the presence of growth factors BDNF, GDNF, and NT3, 6C reprogrammed mouse astrocytes into glutamatergic CiNs expressing Tuj1 and DCX. However, when they added dopaminergic factors (SAg, FGF-8b, TGF-β3, and vitamin C) four days after 6C induction, they did not obtain dopaminergic CiNs. In their work, the authors demonstrated the possibility of reprogramming astrocytes into CiNs without the use of epigenetic modifiers [89].

A mixture of the small molecules VCR (Table 2) or VPA alone activated the expression of the TFs NeuroD1 and NGN2, but this process was restricted to mouse astrocytes and did not induce morphological changes in human astrocytes [96]. A combination of kenpaullone, forskolin, Y-27632, purmorphamine, and retinoic acid reprogrammed human astrocytes and the spinal cord astrocytes of mice with amyotrophic lateral sclerosis (ASL) into motor CiNs in vitro [90]. In addition, recent work has confirmed the possibility of the direct reprogramming of astrocytes into CiNs (without going through the NPC stage) using small molecules that affect signaling pathways. Treatment with the small molecule DAPT, an inhibitor of γ-secretase, by suppressing the production of Notch-ICD, the intracellular domain of Notch signaling, differentiated astrocytes into DCX-positive neuroblasts in 7 days [91]. Researchers have demonstrated a novel strategy to directly transdifferentiate rat endogenous reactive astrocytes into CiNs using ginsenoside Rg1, an inhibitor of the Notch/Stat3 signaling pathway, and to stimulate neuronal regeneration after spinal cord injury [92]. However, the CiNs were obtained by direct reprogramming, bypassing the proliferation stage, so the number of newly formed neurons depended on the initial number of astrocytes, which to some extent limits the possibility of cell therapy [96].

#### 2.3.3. Reprogramming of ESCs and IPSCs into CiNSCs and CiNs

**Figure 5:**
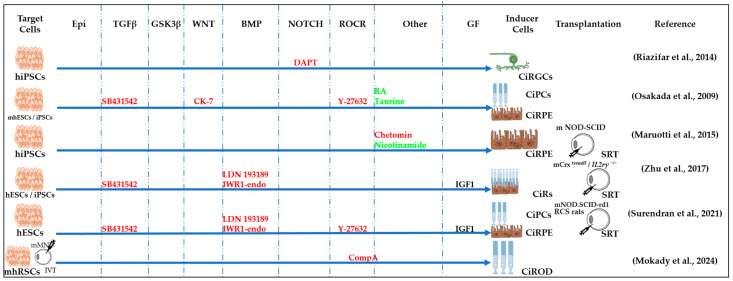
Schematic representation of chemically induced reprogramming of ESCs and iPSCs into neural stem cells and into neurons of the brain and retina [2,47,52,54,97,98,99]. Chemically induced reprogramming utilized small molecules that acted on the cellular epigenome (Epi) and on various signaling pathways that control cellular identity (TGFβ, GSK3β, WNT, BMP, NOTCH, ROCK, and others). GF—growth factor; iPSC—induced pluripotent stem cell; ESCs—embryonic stem cells; RSCs—retinal stem cell; CiRGCs—chemically induced retinal ganglion cells; CiR—chemically induced retina; CiROD—chemically induced rods; CiRPE—chemically induced retinal pigment epithelium; CiPCs—chemically induced photoreceptor-like cells; m NOD-SCID—the nonobese diabetic/severe combined immunodeficient mouse; m Crx^tvrm65^/IL2rγ^−/−^—model of immunosuppressive mouse/retinal degeneration; m NOD.SCID-rd1—the nonobese diabetic/severe combined immunodeficient mouse model of retinal degeneration; RCS rat—rat model of retinal degeneration from Royal College of Surgeons; IVT—intravitreal injection; SRT—subretinal injection; mMNU—mouse model of N-Nitroso-N-methylurea (MNU)-induced retinal degeneration, m—mouse, h—human. Pathway activators: green color; pathway inhibitors: red color. 2.3.4. Reprogramming of the RPE into CiNSCs and CiNs.

Under certain conditions, human ESCs and iPSCs can be specifically reprogrammed into retinal progenitor cells (RPCs), with subsequent differentiation into retinal ganglion cells, amacrine cells, bipolar cells, horizontal cells, and photoreceptors (Figure 5). Thus, it has been shown that a Notch inhibitor, DAPT, alone can stimulate iPSCs’ differentiation into functional chemically induced retinal ganglion cells (CiRGCs) [52]. The combination of SB431542, CK-7, and Y-27632 is sufficient for human ESCs and iPSCs to differentiate into chemically induced retina (CiR) and chemically induced retinal pigment epithelium (CiRPE) [47]. Researchers from Maruotti’s group, having tested 300 small molecule samples, identified chetomin, a dimeric epidithiodiketopiperazine, as a potent RPE inducer, the combination of which with nicotinamide resulted in the efficient differentiation of chemically induced CiRPE cells from iPSCs [97]. A small-molecule-based protocol for generating retinal progenitors and differentiated retinal cell types, including photoreceptors, from human ESCs and iPSCs was developed using IWR1-endo (Dorsomorphin), SB431542, LDN 193189, and insulin-like growth factor 1 (IGF1) [49,98]. The resulting chemically induced retinal progenitor cells (CiRPCs) and CiPCs were tested in multiple host mouse strains with and without retinal degeneration and demonstrated the ability to survive and functionally integrate into the host mouse retina following transplantation [98,99]. Surendran et al. used growth factors and small molecules IGF-1, IWR1, LDN193189, SB431542, and Y27632 to obtain RPE and photoreceptor precursors from iPSCs. In their work, they showed details of how iPSC differentiation mimics the stages of human retinal development in vivo, starting from undifferentiated iPSCs and ending with the formation of mature CiRPE and CiPCs [2].

**Figure 6:**
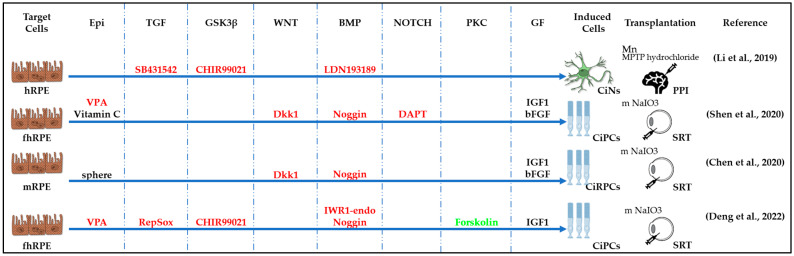
Schematic representation of chemically induced reprogramming of RPE into neural stem cells and into neurons in the brain and retina [12,27,46,93]. Chemically induced reprogramming utilized small molecules that acted on the cellular epigenome (Epi) and on various signaling pathways that control cellular identity (TGFβ, GSK3β, WNT, BMP, NOTCH, PKC, and others). Fh—fetal human; Mn—cynomolgus monkeys (*Macaca fascicularis*); MPTP hydrochloride—induced Parkinson’s disease model; PPI—implantation into posterior putamen; sphere—free floating conditions; for other abbreviations, refer to Figure 3 and Figure 5. Pathway activators: green color; pathway inhibitors: red color.

Interestingly, adult human eye RPE cells can be differentiated into dopaminergic CiNs by a small molecule cocktail consisting of LDN193189, CHIR99021, and SB431542 [93] (Figure 6). Shen et al. used VPA and vitamin C to induce the rejuvenation of human fetal RPE cells into a stem state (fRPESCs) and then directed the differentiation of fRPESCs into CiPCs using a custom three-step protocol based on different culture systems and media. They used Noggin, DAPT, Dkk1, IGF1, and bFGF to stimulate photoreceptor differentiation [46]. During the differentiation, the fRPESCs changed their morphology, forming a rounded shape first and then extending several synapse-like structures to finally form a tubular rod-shaped structure resembling the outer segments of photoreceptors. At the first stage of differentiation, retinal photoreceptor progenitor cells were obtained with the expression of PAX6 and VSX2 markers. At the second stage, the cells differentiated into photoreceptor progenitors that significantly increased the expression of NRL and CRX photoreceptor markers. At the terminal stage of RPESC differentiation, rods with the expression of REC, RHO, ARRESTIN, and GNAT1 were obtained [46]. In another study, it was possible to differentiate mouse RPE cells derived from spheres into cells expressing both the neural markers Tubb3, Rho, and Rec, and the neuroglial marker, Gfap. Among the differentiating cells, no cells immunopositive for PKC-alpha (a marker of ON bipolar cells) or calbindin (a marker of horizontal cells) were detected. Interestingly, the expression of the neural markers βIII-tubulin (Tubb3) and nestin (Nes) and the neuroglial marker Gfap gradually increased, whereas the expression of the early regulators of photoreceptor differentiation, Six6, Pax6, Otx2, Rax, and Nrl, could hardly be detected by qPCR throughout the process of the differentiation of photoreceptors [27].

Deng’s research team used a protocol to chemically reprogram fibroblasts into CiPCs to generate photoreceptor progenitor cells from human fetal RPE cells [12,86]. Noggin and IGF1 in combination with five low-molecular factors (VPA, CHIR99021, RepSox, forskolin, and IWR1-endo) were added to the culture medium. Sonic Hedgehog, taurine, retinoic acid, and bFGF were added to stimulate and support the formation of CiPCs [12]. A gene ontology analysis of the results of the RNA sequencing of these cells revealed upregulation of the genes, such as SOX8, IGFN1, ASCL1, RXRG, THRB, and RORB, involved in neuron generation, neurotransmitter uptake, and photoreceptor differentiation. At day 10 of reprogramming, the transcriptome profile showed robust upregulation of rod-specific, but not cone-specific genes [12]. Studies show that under certain conditions, the RPE is capable of differentiating into photoreceptors, particularly rods.

The chemical compounds retinoic acid and taurine are known to be crucial for the development of photoreceptors and to promote photoreceptor differentiation in the ESC culture system [47,100]. Transient exposure to low doses of retinoic acid (RA) was reported to inhibit ESC differentiation by blocking the canonical Wnt pathway. This treatment results in the preservation of the pluripotency of the ground state of stem cells [101]. Retinoic acid can be used to induce neural differentiation in RPE cells in vitro. The work performed on ARPE-19 and H80HRPE-6 cell lines derived from 19- and 80-year-old donors, respectively, showed that adult human RPE cells are able to differentiate into neurons when exposed to retinoic acid [102]. After stimulation with retinoic acid, RPE cells were stained with antibodies against Map5 and NF200. The proportion of Map5-positive cells was higher in the RPE cell population from a young donor. RPE cells from an old individual were also capable of differentiating into neurons, although the proportion of mature neurons was lower than in the culture of cells from a young donor. Fenretinide, a synthetic derivative of retinoic acid, has a similar effect on RPE cells. Fenretinide-treated ARPE-19 and H1RPE7 cell lines expressed a marker of neural stem/progenitor cells, Pax6, and markers of mature neurons, NF200, NSEγ, calbindin, and calretinin, and the neural cell adhesion molecule, N-CAM. In addition, the cells were found to contain protein markers of photoreceptors (Opn3, Opn4, Nrl, Crx, Opn1mw/1w, SAg, Nr2e3, and Rcvrn) and ganglion cells (Try-1, Brn3, Bag-1), as well as the ganglion cell marker, GFAP [103,104].

After obtaining rejuvenated somatic cells with the features of progenitor cells, it is necessary to start the process of their differentiation in the desired direction. In our case, the factors stimulating neural differentiation in epigenetically rejuvenated epithelial fibroblasts and dedifferentiated RPE are the same [12,86]. The presence of forskolin, noggin, retinoic acid, and a number of growth factors important for neurogenesis (bFGF and IGF) is required for successful and effective reprogramming of RPE cells into neural retinal cells (Figure 6). An RPE cell, like any other terminally differentiated cell, has its own characteristics in the process of chemical reprogramming, which must be taken into account when creating protocols. To date, chemical reprogramming protocols for RPE are similar to reprogramming protocols for other somatic cell types (Table 2).

When developing the chemical reprogramming approach, it was noted that all the effective chemical cocktails in cellular reprogramming included VPA, CHIR99021, and RepSox (or SB431542, or A8301) (Table 2). This cocktail, called VCR, is common in somatic cell reprogramming. It causes the generation of a population of activated cells (or mixed precursors, unstable intermediates) from relatively static somatic cells, regardless of the type of somatic cell and the outcome [42]. This suggests that VPA, CHIR99021, and RepSox have an additive effect and contribute potently to cellular rejuvenation and dedifferentiation. Ma et al. demonstrated that the cocktail without either Forskolin, I-BET151, ISX9, or CHIR99021 failed to generate CiNs both in vitro and in vivo. This result further demonstrated that the generation of CiNs was induced by the synergetic function of each small molecule [88]. Other research groups have observed that chemical cocktails containing CHIR99021 and RepSox can induce direct reprogramming between differentiated cell states [56]. The processes related to both rewriting and replacing cellular epigenetic identity are suggested to be influenced by the additive effects of these chemicals. Moreover, independent studies have found associations with individual chemicals and reprogramming in different contexts, indicating that each component probably promotes rejuvenation through a wide range of mechanisms [56].

## 3. Chemical Therapy for Degenerative Eye Diseases

Another important purpose of regenerative medicine is to support and preserve dying RPE cells and photoreceptors in the development of neurodegenerative eye diseases. However, this approach will be successful only when understanding the development processes of a certain disease and at early stages of its development, when cell loss has not yet led to dramatic consequences. To achieve this goal, molecular complex therapy for hereditary eye diseases based on pharmacological small molecules is actively being developed [105,106]. Depending on the mechanism of action of small molecules, several treatment options can be distinguished: epigenetic therapy; therapy based on regulation of the activity of transcription factors; translational readthrough (TR) therapy; protein therapy; and specific therapy. Small molecules as epigenetic regulators give great hope for the possibility of creating a compound that will have not only a therapeutic effect in the treatment of neurodegenerative eye diseases due to direct cellular reprogramming, but also a preventive effect due to cellular rejuvenation. Thus, it was shown that the small molecules of the VC6TF cocktail that can restore an epigenetic state in cells are able to effectively “rejuvenate” cells. The effect of the drugs will depend on the concentration of the small molecule and the duration of the cell’s exposure to it [58].

In recent years, various strategies based on small molecules have emerged to inhibit transcription of the specific genes involved in cancer, viral infections, and other diseases. TFs require cofactors for their function, and the development of small molecule drugs that interfere with TFs/cofactors or disrupt the homo-/heterodimerization of TF molecules has yielded various highly specific drugs that can neutralize specific DNA-regulatory proteins [107]. Photoregulin 3 (PR3) was shown to interact with photoreceptor cell-specific nuclear receptor Nr2e3 and antagonize its activity through binding to its transcriptional cofactors. Thus, photoreceptor degeneration in the model of RhoP23H mice was successfully prevented, leading to the first report of the successful treatment of retinitis pigmentosa with a small molecule in vivo [108]. JQ1, through targeting BET proteins involved in the regulation of TFs translation, abolishes microglial activation in vitro and in vivo in rd10 retinas and effectively preserves the survival and function of rd10 mouse photoreceptor cells. These results suggest a new paradigm for the treatment of retinitis pigmentosa by targeting epigenetic readers of BET [109].

TR therapy is based on small molecules, also known as TR-inducing drugs (TRIDs), which enable the translation machinery to bypass a premature termination codon during translation. They can induce mRNA degradation through nonsense-mediated decay and thereby inhibit the expression of full-length proteins. The insertion of an amino acid at the site of a premature stop codon can increase full-length protein expression, as well as decreasing nonsense-mediated decay [105,106]. There are two main classes of TRIDs, aminoglycoside and non-aminoglycoside TRIDs, which are being actively studied in various disease models, including inherited eye diseases [105,106]

Protein therapy is based on the administration of drugs that restore proteostasis, such as pharmacological chaperones, kosmotropes, molecular chaperones, or autophagy inducers [105,110]. Thus, the role of the small molecule, arimoclomol, in the restoration of rhodopsin proteostasis was demonstrated [111].

In 2013, Swoboda and colleagues showed that the small molecule, WS3, a diarylurea analogue, promoted the proliferation and survival of human embryonic and adult RPE cells in vitro, and preserved cell functionality in vitro after differentiation and in vivo after subretinal transplantation [53].

The protection of RPE cells from oxidative stress was demonstrated in the experimental ARPE-19 model after exposure to natural and synthetic flavonoids. Flavonoids are a class of natural small molecules that have evolved to protect plants from oxidative damage induced by chronic exposure to ultraviolet light. Effective flavonoids included the dietary flavonoids fisetin, luteolin, quercetin, eriodictyol, baicalein, galangin, and EGCG, as well as the synthetic flavonoids 3,6-dihydroxyflavonol and 3,7-dihydroxyflavonol. The authors showed that some flavonoids could protect RPE cells, even when added after the cells had been exposed to oxidative stress. The flavonoids acted through an intracellular pathway to block the accumulation of reactive oxygen species. Many of these flavonoids induced the expression of NRF2 and phase-2 gene product heme oxygenase-1 in human RPE cells [112].

Vitamin C acts as a scavenger of reactive oxygen species (ROS), which can cause cellular damage and interfere with the reprogramming process. Vitamin C is also a cofactor for enzymes involved in collagen biosynthesis. Thus, vitamin C plays an important role in promoting the generation of iPSCs from somatic cells in both humans and mice by providing an antioxidant environment and by altering epigenetic regulation [33,113].

Vighi and colleagues administered a liposomal cGMP analog, LP-CN03, and demonstrated improvement of visual function and reduction in photoreceptor degeneration in mouse models carrying mutations in various orthologs of the gene for inherited retinal diseases [114]. Taken together, these data suggest that cGMP signaling may be a common pathway for the treatment of genetically and phenotypically distinct types of retinal degeneration [114,115].

Another drug previously tested in clinical trials is a synthetic oral cis-retinoid also known as QLT091001 [105]. This drug induced vision restoration in transgenic and naturally occurring RPE65 mutant mouse and dog models [116]. The results of a clinical trial showed that QLT091001 did improve visual function in patients with inherited retinal diseases associated with LRAT and RPE65 mutations [117].

A number of new small molecules have shown promise for further development. CompA administered via intravitreal injection promoted the regeneration of photoreceptor function in a mouse model of retinitis pigmentosa, which occurred through the increased proliferation, integration, and differentiation of retinal stem/progenitor cells [54]. The authors identified several kinases inhibited by CompA, including p70S6K, ROCK1, and PKG, in vitro. mTOR signaling and ROCK signaling are involved in the regulation of progenitor cell proliferation.

Azobenzene-based photoswitches have also been shown to be effective in restoring vision in animal models of outer retinal degeneration [118]. After the death of photoreceptors in AMD and retinitis pigmentosa, the retina undergoes remodeling, which involves structural and functional changes [118]. Despite many changes in the retina during remodeling, the connections of ganglion cells to the brain remain intact, making researchers speculate that the photosensitization of these cells could restore light perception and, possibly, image-forming vision in blind animals. In this approach, voltage-gated-potassium-channel-blocking drugs become active in the light via photoisomerization of the covalently linked azobenzene moiety [119]. When injected into the vitreous body, these compounds make retinal ganglion cells directly photosensitive [120]. This approach avoids the irreversibility of gene therapy, but requires repeated courses of treatment [13].

The development of small-molecule-based therapy for neurodegenerative eye diseases is only just beginning, but it has already shown great potential. Depending on the goal, to restore or to preserve, a specific set of small molecules can be used. For example, a cocktail containing epigenetic modulators would be promising and effective for the restoration of RPE cells and neural retinal cells via the transdifferentiation (reprogramming) of the endogenous RPE. For the preservation of cells, the same epigenetic regulators or CompA would be necessary. Small molecules that demonstrate a protective effect on RPE cells and photoreceptors can be widely used in various eye diseases. However, to use highly specific small molecules that directly regulate the expression of TFs or translational readthrough, precise and clear understanding of the mechanism of neurodegenerative disease is necessary.

## 4. Conclusions

Direct reprogramming represents a feasible approach for generating cells in vitro for biological research, tissue engineering, and transplantation. Moreover, the application of direct reprogramming in vivo provides an exciting regenerative strategy for reprogramming and reprofiling endogenous cells in damaged tissues, which overcomes the limitations associated with donor cell transplantation. Existing studies show high potential for vertebrate RPE cells to be reprogrammed into retinal neurons in vivo. Although there are few studies on reprogramming human RPE cells, one can safely say that human RPE cells are capable of responding not only to genetic engineering methods but also to molecular therapy. Small molecules do reprogram RPE cells into retinal neural cells in vitro.

Compared with other methods, using small molecules has a number of unique advantages, such as structural versatility and ease of manipulation, depending on the time and concentration. However, one should not forget about the possible drawbacks of this approach, such as toxicity, issues with modes of delivery, and the fact that the process of selecting necessary small molecules can be labor-intensive and expensive. Developing combinations of small molecules that can successfully reprogram the RPE, as well as any other cell type, may require a thorough understanding of the mechanisms of action and kinetics of each compound, which would require significant time for additional screening and the determination of optimal concentrations. In addition, different combinations induce different neuronal properties and provide variations in transformation efficiency, which requires further study [55]. Fang et al., in their work, raised a number of important questions and discussed serious obstacles that need to be overcome before clinical use of the reprogramming techniques [121]. These are the potential risk of endogenous cell depletion; the persistence of genetic mutations in reprogrammed cells and, therefore, a tendency toward cell degeneration in the future; and the identification of reprogramming factors that promote conversion to a certain type of a target cell. Thus, the delivery system for reprogramming factors must be safe, effective, and specific for target cells, and the system for tracking and analyzing cellular reprogramming in vivo must also be developed, which is a long and laborious process [121].

However, today, many researchers believe that the development of technologies using small molecules and the CRISPR/CAS9 system can form the basis for successful regenerative therapy for tissue restoration. Based on the results obtained from reprogramming vertebrate cells into retinal cells and on the achievements of computational biology, it is possible to suggest the most optimal set of epigenetic, genetic, and chemical factors with the addition of neurotropic factors and growth factors for the successful conversion of human RPE cells into retinal neurons.

## Figures and Tables

**Table 1:** Small molecules used for complete chemically induced reprogramming or transdifferentiation of different cell types into neural cells; their chemical structure and proposed mechanism of action.

Small Molecule	Chemical Structure	Function	Induced Cells	References
**Epigenetic Regulators**				
VPA	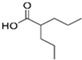	An inhibitor of HDAC	CiNPCs, CiNs	[36,37,38]
NaB	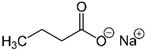	An inhibitor of HDAC	CiNPCs	[36,39]
TSA	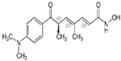	An inhibitor of HDAC	CiNPCs	[36]
SAHA	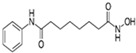	An inhibitor of HDAC I/II	CiNPCs	[40]
RG108	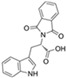	An inhibitor of DNA methyltransferases (DNMTs)	CiNPCs	[41]
Parnate	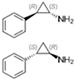	A histone demethylase inhibitor	CiNSCs, CiNs, CiRPCs, CiRNs	[41]
BrdU)	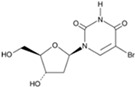	A thymidine nucleoside derivative commonly used to identify proliferating cells	CiPSCs	[42]
EPZ004777	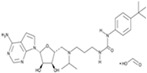	A potent inhibitor of histone H3K79 methyltransferase (DOT1L)	CiPSCs	[42]
SGC0946	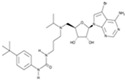	A potent inhibitor of DOT1L	CiPSCs	[42]
DZNep	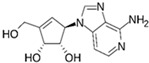	A S-adenosylhomocysteine synthesis inhibitor and a histone methyltransferase EZH2 inhibitor	CiNPCs	[42]
5-aza-dC	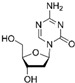	A DNMT inhibitor	CiPSCs	[42]
I-BET151 (GSK1210151A)	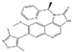	An inhibitor of a BET bromodomain	CiNs	[43]
Molecules acting on signaling pathways				
CHIR99021	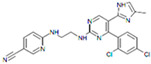	An inhibitor of GSK3	CiNPCs	[36,37,38,39,41,43,44,45,46]
LiCl and Li_2_CO_3_		An inhibitor of GSK3	CiNPCs	[36]
Forskolin	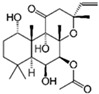	An activator of adenylate cyclase	CiNPCs, CiNs	[37,43]
SB431542	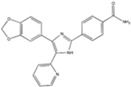	An inhibitor of TGF-βRI	CiNSCs, CiNs, CiRPCs, CiRNs	[36,38,43,44,47,48,49,50]
RepSox (E-616452)	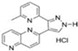	An inhibitor of TGF-βRI (ALK5)	CiNPCs, CiNs	[36,37]
A83-01	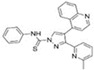	An inhibitor of TGF-βRI (ALK4/5/7)	CiPSCs	[41,42]
Tranilast	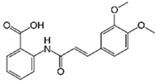	An inhibitor of TGF-β1	CiPSCs, CiNPCs	[36]
LDN193189	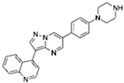	A BMP type I receptor (ALK2/3) inhibitor	CiNSCs, CiRNs	[38,41,49,50]
Dorsomorphin (IWR1-endo)	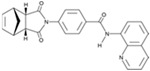	An inhibitor of AMPK and BMP I	CiNs	[37,38,49]
DAPT	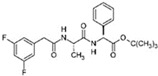	An inhibitor of Notch	CiNs, CiRGCs	[38,50,51,52]
RA	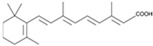	An activator of PI3K	CiNSCs, CiNs, CiRPCs, CiRNs	[41,49]
SMER28	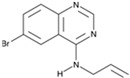	An enhancer of autophagy	CiPSCs	[41]
ISX9	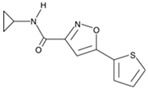	A WNT pathway activator. An inducer of adult neural stem cell differentiation	CiNs	[43]
GO6983	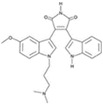	An inhibitor of protein kinase C	CiNs	[37]
SP600125	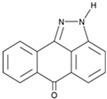	A pan-JNK inhibitor	CiNs	[37,39]
Hh-Ag1,5 (SAg)	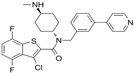	A smoothened (SMO) agonist, Hedgehog signaling pathway activator	CiNPCs	[38,41,50]
Purmorphamine	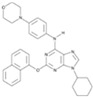	An agonist of SMO, Hedgehog signaling pathway activator	CiNs	[4,38,42]
CKI-7	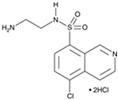	An inhibitor of Wnt and casein kinase 1 (CK1)	CiRNs	[4,47]
IQ-1	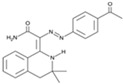	A WNT pathway activator. Binds to PP2A, decreases phosphorylation of p300	CiPSCs	[4]
TWS119	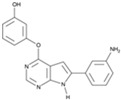	An inhibitor of GSK3β	CiNs	[4]
Pyridium	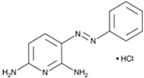	Mode of action unspecified	CiNs	[4]
TTNPB	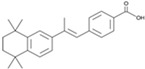	A potent selective RAR agonist	CiPSCs, CiNs	[38,42]
Ch 55	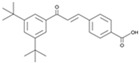	A RAR agonist	CiPSCs	[42]
AM580 (CD336)	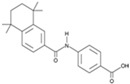	A selective RARα agonist	CiPSCs	[42]
PD0325901	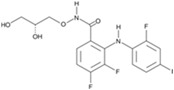	A MEK inhibitor that sustains stem cell renewal	CiPSCs	[42]
LPA	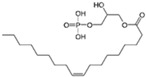	A lipid mediator of several receptors of the GPCR family	CiNSCs	[39]
Rolipram	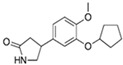	A selective phosphodiesterase-4 inhibitor	CiNSCs	[39]
**Compounds that promote survival, proliferation, and function of reprogrammable cells**				
Y-27632	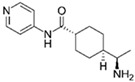	A ROCK inhibitor	CiNs, CiRNs	[37,47,50]
HA-1077	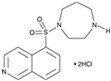	A ROCK inhibitor	CiNs	[43]
SB203580	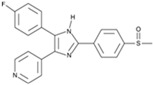	A specific p38 MAPK inhibitor	CiNs	[43]
BIRB796	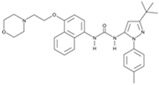	A potent inhibitor of p38 MAPK	CiNs	[43]
WS3	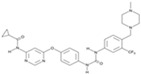	A non-specific proliferative molecule	RPE	[53]
Thiazovivin	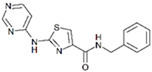	A ROCK inhibitor	CiNs	[38,42]
CompA	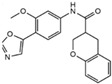	An inhibitor of p70S6K, ROCK1, and PKG	Photorecep-tors	[54]

Note: VPA—valproate (valproic acid); NaB—sodium butyrate; TSA—trichostatin A; SAHA—vorinostat (suberoylanilide hydroxamic acid); RG108—N-Phthalyl-L-tryptophan; BrdU—bromodeoxyuridine (broxuridine); parnate—tranylcypromine; DZNep—3-Deazaneplanocin A; 5-aza-dC—5-Aza-2′-Deoxycytidine; RA—retinoic acid (vitamin A); SMER28—bromo-substituted quinazoline; ISX9—isoxazole 9; pyridium—phenazopyridine; TTNPB—Arotinoid acid; Ch55—a synthetic analog of retinoic acid; AM580 (CD336)—a stable benzoic derivative of retinoic acid; PD0325901—mirdametinib; LPA—lysophosphatidic acid; HA-1077—fasudil; BIRB796—doramapimod; CiPSCs—chemically induced pluripotent stem cells; CiNSCs—chemically induced neural stem cells; CiNs—chemically induced neurons; CiRPCs—chemically induced retinal progenitor cells, CiRNs—chemically induced retinal neurons; CiRGCs—chemically induced retinal ganglion cells; RPE—retinal pigment epithelium.

**Table 2:** Small molecule cocktails used to reprogram somatic cells into brain or retinal neurons.

Cocktail Name	Small Molecules	Functions	Cell Reprogramming	Reference
	Forskolin	An activator of adenylate cyclase	Mouse fibroblast into CiNs	[85]
	Dorsomorphin (IWR1-endo)	An inhibitor of AMPK and BMP I		
	CHIR99021	An inhibitor of GSK3β	Human fibroblast into CiNs	[44]
	SB431542	An inhibitor of TGF-βRI		
	Noggin	An inhibitor of BMP signaling		
VCR	VPA	An inhibitor of HDAC	Mouse fibroblast into CiNPCs	[36]
	CHIR99021	An inhibitor of GSK3β		
	RepSox	An inhibitor of TGF-β (ALK5)		
FICB	Forskolin	An activator of adenylate cyclase	Human fibroblast into CiNs	[43]
	ISX9	An inducer of adult neural stem cell differentiation		
	CHIR99021	An inhibitor of GSK3β		
	SB431542	An inhibitor of TGF-βRI		
FICB+1	Forskolin	An activator of adenylate cyclase	Human fibroblast into CiNs	[43]
	ISX9	An inducer of adult neural stem cell differentiation		
	CHIR99021	An inhibitor of GSK3β		
	SB431542	An inhibitor of TGF-βRI		
	I-BET151	An inhibitor of a BET bromodomain		
VCRFSGYD	VPA	An inhibitor of HDAC	Mouse fibroblast into CiNs	[37]
	CHIR99021	An inhibitor of GSK3β		
	RepSox	An inhibitor of TGF-β (ALK5)		
	Forskolin	An activator of adenylate cyclase		
	Sp600125	An inhibitor of JNK		
	GO6983	An inhibitor of PKC		
	Y27632	An inhibitor of ROCK		
	Dorsomorphin (IWR1-endo)	An inhibitor of AMPK and BMP I		
	CHIR99021	An inhibitor of GSK3β	Mouse fibroblast into CiNs	[38]
	SB431542	An inhibitor of TGF-βRI		
	LDN193189	A BMP type I receptor (ALK2/3) inhibitor		
	TTNPB	An RAR ligand		
	Thiazovivin (Tzv)	An inhibitor of ROCK		
	VPA	An inhibitor of HDAC	Human fibroblast into CiNs	[37]
	DAPT	An inhibitor of Notch via gamma-secretase		
	Dorsomorphin (IWR1-endo)	An inhibitor of AMPK and BMP I		
	SAg	A smoothened agonist (SMO), an activator of Hedgehog		
	NaB	An inhibitor of HDAC	Human fibroblast into iNSCs	[39]
	CHIR99021	An inhibitor of GSK3β		
	A83-01	An inhibitor of TGF-βri (ALK4/5/7)		
	LPA	A lipid mediator of several receptors of the GPCR family		
	Rolipram	An inhibitor of PDE4		
	SP600125	An inhibitor of JNK		
M9	CHIR99021	An inhibitor of GSK3β	MEF into iNSCs	[41]
	LDN193189	A BMP type I receptor (ALK2/3) inhibitor		
	A83-01	An inhibitor of TGF-βri (ALK4/5/7)		
	Retinoic acid	An agonist of RAR		
	Hh-Ag1.5	A smoothened agonist (SMO), an activator of Hedgehog		
	RG108	An inhibitor of DNMT		
	Parnate	A histone demethylase inhibitor		
VCRFD	VPA	An inhibitor of HDAC	Fibroblast into CiNs	[86]
	CHIR99021	An inhibitor of GSK3β		
	RepSox	An inhibitor of TGF-β (ALK5)		
	Forskolin	An activator of adenylate cyclase		
	Dorsomorphin (IWR1-endo)	An inhibitor of AMPK and BMP I		
	VPA	An inhibitor of HDAC	Human astrocytes into CiNs	[38]
	CHIR99021	An inhibitor of GSK3β		
	SB431542	An inhibitor of TGF-βRI		
	LDN193189	A BMP type I receptor (ALK2/3) inhibitor		
	DAPT	An inhibitor of Notch via gamma-secretase		
	TTNPB	A ligand of RAR		
	Tzv	A Rho/rock pathway inhibitor		
	SAg	A smoothened agonist (SMO), an activator of Hedgehog		
	Purmorphamine	A smoothened agonist (SMO), an activator of Hedgehog		
VCR	VPA	An inhibitor of HDAC	Mouse astrocytes into CiNs	[34]
	CHIR99021	An inhibitor of GSK3β		
	RepSox	An inhibitor of TGF-β (ALK5)		
VCRFI + 1	VPA	An inhibitor of HDAC	Human astrocytes into CiNs	[87]
	CHIR99021	An inhibitor of GSK3β		
	RepSox	An inhibitor of TGF-β (ALK5)		
	Forskolin	An activator of adenylate cyclase		
	i-BET151	An inhibitor of a BET bromodomain		
	ISX9	An inducer of adult neural stem cell differentiation		
FICY + 1	Forskolin	An activator of adenylate cyclase	Human astrocytes into CiNs	[88]
	ISX9	An inducer of adult neural stem cell differentiation		
	CHIR99021	An inhibitor of GSK3β		
	i-BET151	An inhibitor of a BET bromodomain		
	DBcAMP	A phosphodiesterase inhibitor		
	Y-27632	An inhibitor of ROCK		
6C	SAg	A smoothened agonist (SMO), an activator of Hedgehog	Astrocytes into CiNs	[89]
	CHIR99021	An inhibitor of a GSK3β		
	DAPT	An inhibitor of Notch via gamma-secretase		
	Ruxolitinib	An inhibitor of JAK 1 and JAK 2		
	RepSox	An inhibitor of TGF-β (ALK5)		
	Y-26732	An inhibitor of ROCK		
	Kenpaullone	An inhibitor of GSK 3α and GSK 3β	Human and mouse astrocytes into CiNs	[90]
	Forskolin	An activator of adenylate cyclase		
	Y-27632	An inhibitor of ROCK		
	Purmorphamine	A smoothened agonist (SMO), an activator of Hedgehog		
	Retinoic acid	A RAR ligand, activates PI3K		
	VPA	An inhibitor of HDAC	Human astrocytes into CiNs	[90]
	RepSox	An inhibitor of TGF-β (ALK5)		
	SB431542	An inhibitor of TGF-βRI		
	DAPT	An inhibitor of Notch via gamma-secretase		
	Dorsomorphin (IWR1-endo)	An inhibitor of AMPK and BMP I		
	LDN193189	A BMP type I receptor (ALK2/3) inhibitor		
	CHIR99021	An inhibitor of GSK3β		
	DAPT	An inhibitor of Notch via gamma-secretase	Mouse astrocytes into CiNs	[91]
	Ginsenoside Rg1	An inhibitor of Notch	Rat astrocytes into CiNs	[92]
	SB431542	An inhibitor of TGF-βRI	Mouse iPSCs into CiRGCs and CiRPE	[47]
	CK-7	An inhibitor of Wnt		
	Y-27632	An inhibitor of ROCK		
	SB431542	An inhibitor of TGF-βRI	Human ESCs/iPSCs into CiRNs, including CiPCs	[49]
	LDN 193189	An inhibitor of BMP type I (ALK2/3)		
	Dorsomorphin (IWR1-endo)	An inhibitor of AMPK and BMP I		
CBL	CHIR99021	An inhibitor of GSK3β	Human RPE into CiNs	[93]
	SB431542	An inhibitor of TGF-βRI		
	LDN193189	A BMP type I receptor (ALK2/3) inhibitor		
VV	VPA	An inhibitor of HDAC	Human RPE into CiPCs	[46]
	Vitamin C			
	DAPT	An inhibitor of Notch via gamma-secretase		
VCRFD	VPA	An inhibitor of HDAC	Human RPE into CiPCs	[12]
	CHIR99021	An inhibitor of GSK3β		
	RepSox	An inhibitor of TGF-β (ALK5)		
	Forskolin	An activator of adenylate cyclase		

Note: CiNSCs—chemically induced neural stem cells; CiNs—chemically induced neurons; MEF—mouse embryonic fibroblast; ESCs—embryonic stem cells; iPSCs—induced pluripotent stem cells; iNSCs—induced neural stem cells; CiRNs—chemically induced retinal neurons; CiRGCs—chemically induced retinal ganglion cells; CiRPE—chemically induced retinal pigment epithelium; RPE—retinal pigment epithelium; CiPCs—chemically induced photoreceptor-like cells.

## Data Availability

Not applicable.
